# Iowa State Veterinary Medical Association

**Published:** 1894-12

**Authors:** 


### SOCIETY PROCEEDINGS.

### THE IOWA STATE VETERINARY MEDICAL ASSOCIATION.

The Iowa State Veterinary Medical Association held its seventh annual meeting at the Savery Hotel, Des Moines, on November 15th and 16th.

The meeting was called to order by the President, Dr. W. B. Niles, at 10 A. M. Roll call by the Secretary, Dr. J. E. Brown, showed about twenty members present, consisting of Drs. Brown, Edwards, Heck, Hammond, Austin, Johnson, Morse, Lincoln, Niles, Miller, Paine, Stalker, Sayers, Starkey, Stewart, and others. Drs. Adams, Day, Geddes, White, McCarthy, McCall, Peters, Wake, and a few others, were present as visitors. Letters of regret were received from Dr Kennedy, M.D., of Des Moines, Secretary of the State Board of Health ; from Dr. W. L. Williams, of Montana, and from two or three others whose names are not now at hand.

The President, in his annual address, called attention to several things seemingly of importance to the advancement of the profession. The following extracts show the tenor of the paper:

"Our Secretary has truly said in his circular letter, 'The Veterinarian of all others should be a Sanitarian.' How many of us in the past have paid much attention to sanitary medicine ? Very few indeed. It is true that but few people ask for sanitary advice. Is not this condition largely the fault of the Veterinarian ? As he in many cases is not prepared to give such advice, and has not impressed his patrons as having a knowledge of sanitary medicine, is it any wonder that he is not thought of in connection with such matters ?

"The future Veterinarian *must be* a sanitarian. Of all medical men he is best fitted for a sanitary officer ; not only to guard the health of animals, but the public health as well ; being more familiar than the human physician with comparative medicine, he better understands the animal plagues which are communicable to man, and thus better understands the laws necessary for their prevention. The question of sanitation is exceedingly important, not only from an economical standpoint, but on account of the danger to the public health. Tuberculosis in cattle has recently been shown alarmingly prevalent in many States, and well merits the attention it is now receiving. In this State, the disease is, without a doubt, much more prevalent than is usually supposed, and should receive the consideration of all medical men, and the attention of all who are interested in our live stock interests and the health of the people.

"With the improvement in the value of horses which has already been manifested, and the widening field of the veterinarian's work as shown by the increasing demand for meat inspectors, city veterinarians, and other sanitary officers, we sincerely believe that the veterinarian capable of taking his proper position in the field of medicine has a good future before him. It seems to us that the demands for veterinarians with a more broad and liberal education—men capable of taking the advanced positions of the near future—should be a lesson for some of our colleges to enlarge their curriculums and in other ways improve their facilities. It is with some alarm that we view the starting of so many new colleges, which must, judging by the length of the college course, turn out "horse doctors" rather than skilled scientific veterinarians.

"Have we not more to fear from the poorly educated man with his diploma, than from the non-graduate? Many think we have. Would it not be well for us, as an Association, to raise our requirements for membership as high as those of the U. S. V. M. A., and thus stand hand in hand with that society in resisting this threatened invasion of empyricism?

"Would it not also be well to put ourselves on record concerning army legislation? Last spring I wrote many of our Congressmen urging their support of the bill then in the hands of the Military Committee, and from most of them I received very encouraging replies. If we pass a resolution favoring such a bill, it may have some weight. If all the State Associations would do this, it would surely help. Let us do our part."

The printing of the proceedings of the meeting by the Association was also advised.

The Secretary and Treasurer in his report also called attention to some of the questions discussed by the President, urging their importance. His report showed the Association in a prosperous condition financially and otherwise, the Society having about seventy members in good standing.

The Board of Censors reporting favorably on the eighteen applicants for membership, they were duly elected. Many of these were present and took part in the meeting. The attendance of old members and the number of veterinarians joining was noted as being greater than for many years past.

The Committee on Legislation presented an instructive report explaining why desired legislation had not been secured. Although the bill introduced was only intended to regulate the title of the veterinarian, prohibiting non-graduates from assuming the title of the graduate, it could not be passed without amendments which the committee could not agree to; consequently, preferring no legislation to the passage of such an amended bill, it was not reported from the House Committee to which it had been referred. It was found that many members of the Assembly feared the influence of the non-graduates in their localities, and could not be induced to favor any bill which seemed to prevent them from practicing. It will thus be seen that legislation failed largely from lack of influence.

The Committee on Collective Statistics made a brief report, stating some of the difficulties encountered in collecting satisfactory statistics, but recommending that the work be continued for another year, so that the results might be compiled and thus made as valuable as possible.

Committee on securing the 1895 meeting of the U. S. V. M. A. reported that correspondence with Dr. Hoskins, the President of that Association, revealed that the meeting would, in all probability, come west, and that any steps taken for securing it for Des Moines should be taken soon. Correspondence with a number of western members showed they were heartily in favor of holding the meeting in Iowa.

The Association, acting on this report, decided to take active measures to secure the meeting, and appointed a committee of three to undertake the work. An assessment of $2.50 each was levied to constitute an entertainment fund in case the meeting should be secured and the money needed.

Under the head of New Business, several interesting questions were discussed, and, as suggested in President's address, Committees on Sanitation, Publication, Army legislation, and to consider advisability of preparing articles for the Agricultural press, were appointed. Notice of an amendment to the constitution, raising requirements for membership as high as those of the U. S. V. M. A., to be acted upon at next meeting, was given.

The proceedings so far described consumed the time of the morning and afternoon sessions. At the evening session, beginning at 8 P. M., two valuable papers were read. one on "Meat Inspection at the Abattoirs," by Dr. S. Stewart, Government meat inspector of Kansas City, and the other by Dr. G. A. Johnson, City meat inspector of Sioux City, Iowa, on "Meat Inspection at the City Market." Dr. Stewart's paper was illustrated by stereopticon views and described fully the character of Government meat inspection. The kinds of diseased meats most frequently encountered was emphasized and many other points of importance touched upon. The paper was both interesting and instructive, and shed much light on a subject at present little understood by a majority of American veterinarians.

Dr. Johnson's paper was also a valuable contribution to sanitary literature. It gave the ordinance of Sioux City providing for the inspection of meats, fruits, etc., and mentioned the utter impossibility of the ordinance being carried out to the letter. The modus operandi. as carried out by the writer, was described An opinion as to what City inspection should consist of was also given. Both of the papers on meat inspection were very generally discussed, with much profit.

At the morning session on the 16th, about thirty members were present. Dr. W. B. Niles, of the Veterinary Department of the Iowa State College of Agriculture and Mechanic Arts, read a paper on "Milk and Dairy Inspection." This was illustrated by showing views of some of the bacteria which find their way into milk, and also of two tuberculous milch cows, apparently (judging by their appearance) in the best of health. In this paper, the inspection of milk, as carried out in Iowa, was described, and reasons given why a more careful and extended inspection should be made; together with what, in the opinion of the writer, this should consist of. On account of its importance, the subject of Tuberculosis was given prominence, and the results of personal work with the Tuberculin test given.

After a general discussion of this paper, hinging largely on Tuberculosis and the Tuberculin test, Dr. J Miller, of Ottumwa, read an interesting paper on "Diseases of the Liver, Symptoms and Treatment." In this paper the writer described concisely a number of cases of liver disease coming under his observation during several years' practice; symptoms and treatment were made prominent. Quite a discussion followed, some thinking that if Dr. Miller had diagnosed *his* cases correctly, *they* had overlooked the disease in their own practice.

In a paper on "Flatulent Colic and treatment with Trochar and Canula," Dr. Austin, of Newton, Iowa, described puncturing of the colon through the rectal wall. Cases were mentioned where efforts to relieve by puncturing through the flank failed, but relief was quickly afforded by rectal puncture. Discussion brought out the fact that repeated puncturing through the flank was not necessarily serious, and that abscesses did not often occur if instruments were sterilized before using.

Dr. A. B. Morse, of Des Moines, described as "An interesting experiment" a case in which string halt, caused by "tibial neurotomy," was subsequently cured by Peroneo-phalangeal tenotomy. A general discussion of operations for relief of bone-spavin lameness followed.

Dr. C. M. Day, of the Veterinary Department of the Iowa State College of Agriculture and Mechanic Arts, read a paper on "Some Parasites of the Horse." The writer stated that the practitioner, in his opinion, should know more than is generally known of parasites coming under his observations in daily practice. The paper dealt with a few species obtained principally from animals destroyed at the College for dissecting purposes. Owing to lateness of the hour discussion was rather brief.

A voluntary paper was offered by Dr. A. R. Wake, of the Veterinary Department of the Iowa Agricultural College, on the "Tuberculin test for Bovine Tuberculosis." In this the agent tuberculin was described, together with the modus operandi of its use, as carried out in quite a series of tests in which the writer had assisted. The reaction obtained in some cases, what should be considered a reaction, and the reliability of the test, were points discussed. As the subject of Tuberculosis had been extensively discussed before, discussion following this paper was not lengthy.

Election of officers resulted as follows:–President, J. Miller, of Ottumwa; Secretary and Treasurer, J. E. Brown, Oskaloosa; Vice-President, W. H. Austin, Newton.

Committee on resolutions reported several, all of which were adopted unanimously. The most important of these was one calling attention to the prevalence of tuberculosis and urging the necessity of more efficient sanitary regulations.

Before adjourning it was decided to meet at the call of the President and Secretary. This closed one of the most, if not *the* most, successful meetings in the history of the Association. This result is extremely gratifying, especially to those who were instrumental in making the meeting a success. While there is much work for the Association during the coming year, we believe the enthusiasm of the members will insure its accomplishment.

---

